# Interplay between uromodulin excretion and water balance in the general adult population

**DOI:** 10.14814/phy2.70844

**Published:** 2026-03-29

**Authors:** David A. Jaques, Théodore Pasquier, Menno Pruijm, Daniel Ackermann, Murielle Bochud, Olivier Devuyst, Belen Ponte

**Affiliations:** ^1^ Division of Nephrology and Hypertension Geneva University Hospitals Geneva Switzerland; ^2^ Division of Internal Medicine Geneva University Hospitals Geneva Switzerland; ^3^ Division of Nephrology and Hypertension University of Lausanne and University Hospital of Lausanne Lausanne Switzerland; ^4^ Division of Nephrology and Hypertension Inselspital Bern Switzerland; ^5^ Department of Epidemiology and Health Systems University Center for Primary Care and Public Health (Unisanté) Lausanne Switzerland; ^6^ Department of Physiology, Mechanisms of Inherited Kidney Disorders University of Zurich Zurich Switzerland

**Keywords:** copeptin, urine osmolality, uromodulin, vasopressin, water balance

## Abstract

Uromodulin is a kidney‐specific protein that is excreted in the normal urine. Factors regulating its excretion are unknown. Conflicting studies suggest that water balance may influence uromodulin excretion which, in turn, may cooperate with vasopressin to increase water reabsorption in the distal nephron. We analyzed the interplay between uromodulin excretion and water balance in a healthy adult population. Participants were recruited in a population‐based study in Switzerland. The urine uromodulin excretion rate (UUER) (mg/24 h) was measured (ELISA) in parallel with urine osmolality and serum copeptin (pmol/L) (immune‐luminometric assay) as a surrogate for vasopressin. Mixed multi‐variate linear regression models were used. We included 937 participants with 497 (53.0%) women, median age 47.7 years (32.4–61.6) and 27 (2.8%) with eGFR <60 mL/min/1.73m^2^. In univariate analysis, UUER was positively associated with urine volume (*p* < 0.001) and negatively with serum copeptin concentration (*p* < 0.001). In fully adjusted analysis, UUER remained positively associated with urine volume (*p* = 0.004) but not with serum copeptin concentration (*p* = 0.224). In univariate analysis, urine osmolality was negatively associated with UUER (*p* = 0.016). In fully adjusted analysis, namely accounting for serum copeptin and urine volume, urine osmolality was positively associated with UUER (*p* = 0.001). These results indicate that, in a healthy population, urine uromodulin excretion strongly associates with urine volume and, once accounting for confounding factors, influences urine osmolality in addition to vasopressin/copeptin levels. These data substantiate the role of urine flow in regulating uromodulin excretion and suggest an additive effect of uromodulin on urine concentration, alongside vasopressin.

## 1 INTRODUCTION

Uromodulin is the most abundant protein found in the urine of healthy subjects (Devuyst et al., 2017). It is exclusively produced by the tubular cells lining the thick ascending limb (TAL) of the loop of Henle and the initial part of the distal convoluted tubule (DCT). Uromodulin is a GPI‐anchored protein targeted to the apical membrane of the tubular cells, where it is released in the urine by proteolytic cleavage (Weiss et al., 2020). Once in the urine, the uromodulin monomers assemble to form polymeric filaments that aggregate uropathogens and form the matrix of urinary casts (Weiss et al., 2020). Studies in cell systems and transgenic mice substantiated the roles of uromodulin in regulation of blood pressure and innate immunity as well as in risk mitigation of kidney stones and urinary tract infections (Mo et al., 2004, 2007; Trudu et al., 2013). At the epidemiological level, genome‐wide association studies conducted in the general population associated variants in the *UMOD* gene with an increased risk of developing chronic kidney disease (CKD) and hypertension (Köttgen et al., 2009; Padmanabhan et al., 2010).

Despite these advances, the physiological determinants of urine uromodulin excretion remain largely unknown. A population‐based study has shown that the 24 h urine uromodulin excretion rate (UUER) is positively associated with kidney size as well as estimated glomerular filtration rate (eGFR) and negatively with age and diabetes (Pruijm et al., 2016). Interestingly, UUER was positively correlated with urine volume in this study (Pruijm et al., 2016). In agreement with these findings, water loading and subsequent increased urine flow were associated with higher UUER in healthy controls as well as stone forming participants (LaFavers et al., 2023). Conversely, in mouse models, water deprivation upregulated uromodulin production and administration of the vasopressin analogue desmopressin increased UUER (Nanamatsu et al., 2021; Takata et al., 2022). Overall, these discordant findings raise the issue of the potential link between water balance, vasopressin, urine volume, and uromodulin excretion.

The concentration of the urine ultimately depends on the osmotic gradient established by the TAL as well as the water permeability of the collecting duct (CD), which is mediated by the vasopressin‐mediated transcription, phosphorylation and membrane trafficking of the water channel aquaporin‐2 (AQP2) (Nielsen et al., 1999). Among its biological functions, uromodulin notably regulates the activity of cotransporter Na+‐K+‐2Cl‐ (NKCC2) in the TAL (Mutig et al., 2011). Accordingly, mice knock‐out for uromodulin show impaired urinary concentrating ability upon water restriction while decreased urine concentration is an early and consistent manifestation of defective uromodulin processing in autosomal dominant tubulointerstitial kidney disease (ADTKD) (Bachmann et al., 2005; Bollée et al., 2011). Once released in the urine and organized into filaments, uromodulin may interact with transport systems located in the apical membrane of tubular cells downstream of the TAL. In particular, uromodulin enhances the amount of phosphorylated AQP2 in the apical membrane of CD cells in vivo (Takata et al., 2022). As the osmotic gradient generated by the TAL cells represents the driving force for water reabsorption in the CD, uromodulin could be implicated in a crosstalk between the TAL and downstream segments, as part of a regulation loop favoring conservation of free water.

Here, we used a large population‐based cohort to shed light on the real‐life interplay between uromodulin excretion, water balance, vasopressin secretion as well as urine volume and concentration. While accounting for serum copeptin (an established surrogate for vasopressin) and urine volume, we analyzed the determinants of UUER and tested the potential association between UUER and urine concentration (Morgenthaler et al., 2006).

## 2 MATERIALS AND METHODS

### 2.1 Participants and study design

The present study was based on a Swiss family population‐based study: the Swiss Kidney Project on Genes and Hypertension (SKIPOGH) study. In SKIPOGH, individuals from the general population in the cities of Bern, Lausanne, and Geneva were randomly selected between 2009 and 2012 as previously described (Ponte et al., 2014). Inclusion criteria were (i) minimum 18 years old, (ii) European ancestry, and (iii) at least one first‐degree family member willing to participate. Exclusion criteria were (i) pregnant or (ii) breastfeeding women.

### 2.2 Measurements and definitions

A single study visit was performed after an overnight fast and included a questionnaire, a complete physical examination, blood sampling, a 24‐h urine collection as well as a kidney ultrasonography. Hematology variables, electrolytes and standard chemistry panel were measured in local university laboratories using standard laboratory methods. Creatinine‐based eGFR was calculated using the 2009 Chronic Kidney Disease Epidemiology Collaboration (CKD‐EPI) and expressed as mL/min/1.73m^2^ (Inker et al., 2012). Plasma osmolality was calculated as 2*(sodium + potassium) + urea + glucose. All variables were entered in mmol/L and result was expressed in mOsm/kg. Urine uromodulin concentration was measured by an in‐house ELISA in 24‐h urine collections as previously described (Youhanna et al., 2014). Human uromodulin (stock solution 100 ug/mL; Millipore) was used for the standard curve. The uromodulin ELISA has a sensitivity of 2.8 ng/mL, a linearity of 1.0, an inter‐assay variability of 3.3% and an intra‐assay variability of 5.5%. Uromodulin was expressed as UUER in mg/24 h. Serum copeptin was measured on −80° frozen EDTA‐plasma samples in a batch using a previously described sandwich immune‐luminometric assay (Thermo Fisher Scientific CT‐proAVP Kryptor; Brahms GmbH, Hennigsdorf, Germany) (Morgenthaler et al., 2006). The lower detection limit was 0.9 pmol/L and the functional assay sensitivity (20% inter‐assay coefficient of variation) was <2 pmol/L. Copeptin was expressed as serum copeptin concentration in pmol/L. Urine osmolality was measured using an Advanced Osmometer (Advanced Instruments, Norwood, MA) based on the freezing‐point depression in 24‐h urine collections. A control (Clinitrol 290) and a set of calibration standards (50, 850, and 2000 mOsm/kg) were used before running of each sample batch. The coefficient of variability was 0.19% in urine. Urine osmolality was expressed in mOsm/kg. Kidney ultrasonography consisted in a gray‐scale B‐mode exam according to a standardized procedure (Ponte et al., 2014). CKD was defined as eGFR <60 mL/min/1.73m^2^. Diabetes was defined as fasting blood glucose ≥7 mmol/L or presence of related medication. Hypertension was defined as office BP ≥140/90 mmHg or presence of related medication.

### 2.3 Statistical analysis

Continuous variables were expressed as median and interquartile range. Categorical variables were expressed as number and relative frequencies (%). Normality of distribution was assessed graphically. Outliers were a priori defined based on 99th percentiles on both sides of distribution for the following variables: urine creatinine excretion (mg/kg/24 h), UUER (mg/24 h), serum copeptin concentration (pmol/L), urine volume (mL/24 h) as well as urine osmolality (mOsm/24 h) (Aguinis et al., 2013). Variables were compared between tertiles of UUER using one‐way ANOVA or Kruskall–Wallis test (depending on result of Bartlett test) for continuous variables and Chi2 for categorical variables. Key variables were transformed to approach normal distribution when necessary: Serum copeptin and urine volume were log‐transformed, while UUER and urine osmolality were square root transformed. Multilevel mixed effect linear regression models were applied to account for the inter‐dependence of family clusters. Family identification was considered as the grouping variable and random effect was applied to the intercept. Every regression model was adjusted for study center as a fixed effect. Additionally, multivariate adjustment for potential confounders was a priori specified and included the following variables in fully adjusted models: Age, gender, diabetes, eGFR, kidney volume, and presence of diuretics. Interaction was tested between key variables based on *p* value for interaction term. Collinearity was tested between key variables using variance inflation factors. Normality of the residuals and homoscedasticity were tested graphically. Sensitivity analyses were a priori specified as follows: (i) further adjustment for urine creatinine excretion (mg/kg/24 h), (ii) further adjustment for urine sodium excretion (mmol/24 h), (iii) exclusion of participants with CKD as defined by eGFR <60 mL/min/1.73m^2^, (iv) use of calculated urine osmolality instead of measured urine osmolality, and (v) inclusion of participants a priori considered as outliers. Data were considered to be missing completely at random, and patients with any missing value on included covariates were excluded from the multi‐variate models. For every model, results are presented as *β* coefficients and associated 95% confidence interval (CI) as well as *p* values. A two‐sided *p* value <0.05 was considered significant in every analysis. Statistical analyses were conducted using STATA version 17 (StataCorp, 4905 Lakeway Drive, College Station, TX 77845). The code underlying the analyses presented in this study is available at: https://doi.org/10.26037/yareta:zwm4np4jxjcifozhzdo6fntlgy.

### 2.4 Ethics

Institutional ethical committees of each participating university hospitals approved the SKIPOGH study (references 091/09 for Bern, 92/07 for Lausanne and 09‐089 Geneva). Every included patient provided written informed consent. This study was conducted according to the declaration of Helsinki.

## 3 RESULTS

### 3.1 Descriptive analysis

The complete SKIPOGH cohort included 1′128 participants. Among those, 84 had missing values on urine uromodulin and/or serum copeptin, while 17 had missing values on any a priori considered covariates. Finally, 90 participants were considered as outliers according to the a priori specified criteria defined in the methods section, leaving 937 participants included in the present analysis (Figure S1).

We included 497 (53.0%) women with median age 47.7 (32.4–61.6) and only 27 (2.8%) participants with CKD. Median UUER was 40.4 (28.9–56.2) mg/24 h. Participants' characteristics are described according to tertiles of UUER in Table 1. With increasing UUER tertiles, participants were younger, less frequently diabetics, had higher eGFR, lower prevalence of CKD, lower urine osmolality, higher urine volume, lower serum copeptin concentration, as well as higher kidney length and volume. Other characteristics were similar between tertiles of UUER.

**TABLE 1:** Characteristics of participants according to tertiles of UUER (mg/24 h) (*N* = 937).

	Tertile 1 (*N* = 313)	Tertile 2 (*N* = 312)	Tertile 3 (*N* = 312)	*p* Value
UUER (mg/24 h)	24.0 (17.6–28.9)	40.4 (36.5–44.6)	61.1 (56.2–72.3)	**<0.001**
Min – max values (mg/24 h)	5.6–32.5	32.6–49.1	49.2–107.7	**<0.001**
Clinical data				
Age (years)	51.6 (33.0–66.8)	46.7 (32.2–59.6)	46.3 (31.3–56.6)	**<0.001**
Gender (women)	173 (55.2%)	172 (55.1%)	152 (48.7%)	0.173
BMI (kg/m^2^)	24.6 (21.5–27.8)	24.3 (21.8–27.1)	24.0 (21.9–27.1)	0.638
Diabetes^a^	27 (8.6%)	9 (2.8%)	5 (1.6%)	**<0.001**
Hypertension^b^	84 (26.9%)	64 (20.5%)	62 (19.8%)	0.065
Diuretics	21 (6.7%)	13 (4.1%)	14 (4.4%)	0.291
SBP (mmHg)	116 (105–128)	115 (105–128)	115 (105–125)	0.210
DBP (mmHg)	74 (67–81)	75 (69–81)	76 (69–82)	0.210
Laboratory data				
eGFR (mL/min/1.73 m^2^)	94.2 (79.5–106.4)	97.6 (86.0–108.4)	100.0 (89.3–110.2)	**<0.001**
CKD^c^	20 (6.3%)	5 (1.6%)	2 (0.6%)	**<0.001**
Plasma osmolality^d^ (mOsm/kg)	299 (195–303)	299 (295–302)	299 (295–303)	0.717
Urine osmolality^e^ (mOsm/kg)	526 (369–689)	493 (356–649)	460 (354–604)	**0.035**
Urine volume (mL/24 h)	1350 (1000–1800)	1585 (1200–2149)	1906 (1400–2378)	**<0.001**
Serum copeptin (pmol/L)	4.2 (2.9–6.6)	3.8 (2.7–5.4)	3.5 (2.4–5.1)	**<0.001**
Ultrasonography data				
Kidney length (mm)	109 (103–114)	109 (103–115)	111 (105–117)	**0.001**
Kidney volume (mL)	126 (107–148)	129 (107–158)	136 (112–165)	**0.001**

*Note*: Bold values correspond to *p* value < 0.05.

Abbreviations: BMI, body mass index; CKD, chronic kidney disease; DBP, diastolic blood pressure; eGFR, estimated glomerular filtration rate; SBP, systolic blood pressure; UUER, urine uromodulin excretion rate.

^a^ Defined as fasting blood glucose ≥7 mmol/L or presence of related medication.

^b^ Defined as office BP ≥140/90 mmHg or presence of related medication.

^c^ Defined as eGFR <60 mL/min/1.73m^2^.

^d^ Calculated as 2×(sodium + potassium) + urea + glucose.

^e^ Measured as described in the methods section.

In univariate analysis, plasma osmolality was positively associated with serum copeptin concentration (*β* = 0.01, 95% CI 0.01–0.02, *p* < 0.001) (Figure S2a). In turn, serum copeptin concentration was positively associated with urine osmolality (*β* = 4.13, 95% CI 3.69–4.58, *p* < 0.001) (Figure S2b) and negatively associated with urine volume (*β* = −0.26, 95% CI −0.30 to −0.22, *p* < 0.001) (Figure S2c).

### 3.2 Determinants of urine uromodulin excretion

We first analyzed the determinants of UUER while accounting for serum copeptin and urine volume (Table 2).

**TABLE 2:** Determinants of UUER^a^ (mg/24 h) in univariate, partially adjusted as well as fully adjusted models (*N* = 937).

	Univariate models		Partially adjusted model^b^		Fully adjusted model^b^		
	*β* coefficient	*p* Value	*β* coefficient	*p* Value	*β* coefficient	95% CI	*p* Value
Urine volume^c^ (mL/24 h)	1.24	**<0.001**	0.83	**0.006**	0.84	0.27 to 1.41	**0.004**
Serum copeptin^c^ (pmol/L)	−0.37	**<0.001**	−2.05	0.159	−1.71	−4.46 to 1.04	0.224
Age (per 10 years)	−0.13	**<0.001**	–		−0.05	−0.13 to 0.02	0.168
Gender (women)	−0.22	**0.017**			−0.20	−0.41 to 0.00	0.056
Diabetes (yes)	−0.99	**<0.001**			−0.81	−1.24 to −0.38	**<0.001**
eGFR (per 10 mL/min/1.73m^2^)	0.15	**<0.001**			0.08	0.00 to 0.16	**0.032**
Kidney volume (per 100 mL)	0.52	**<0.001**			0.43	0.15 to 0.72	**0.003**
Diuretics (yes)	−0.14	0.504			0.24	−0.15 to 0.64	0.229

*Note*: All models are adjusted for center as a fixed effect and family as a random effect (not shown). Bold values correspond to *p* value < 0.05.

Abbreviations: eGFR, estimated glomerular filtration rate; UUER, urine uromodulin excretion rate.

^a^ Square root transformed.

^b^ Interaction between urine volume and serum copeptin is considered (not shown, see text).

^c^ Log transformed.

**FIGURE 1:**
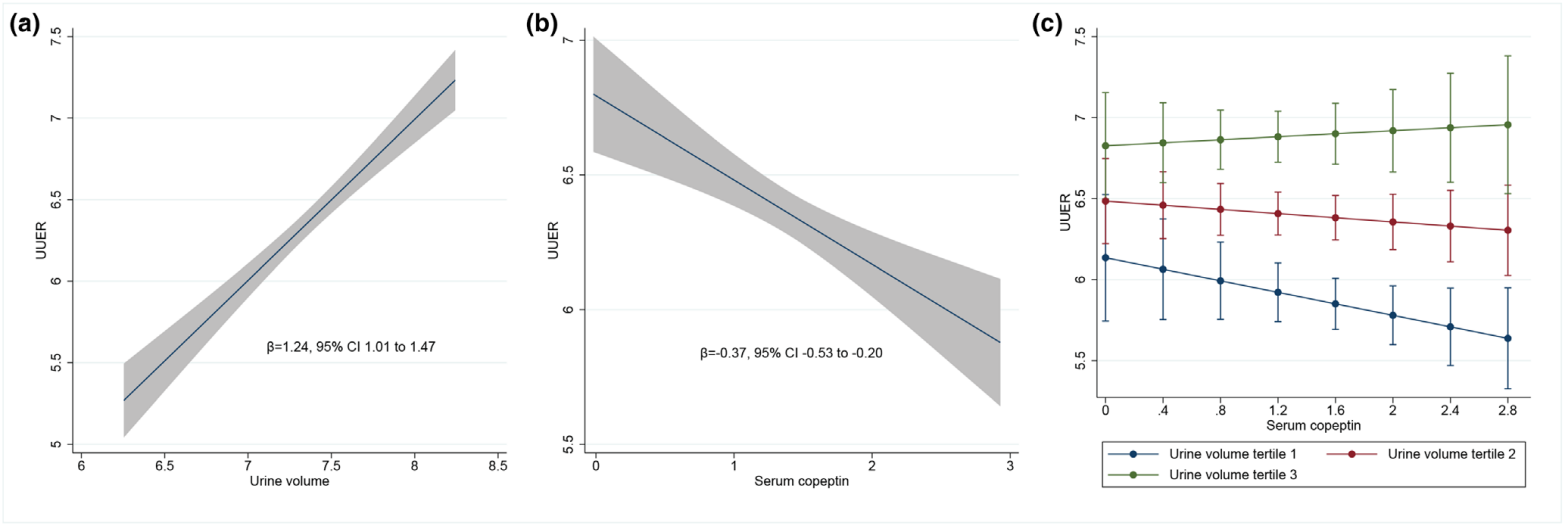
Determinants of UUER (mg/24 h) in distinct univariate models and single partially adjusted model (*N* = 937). (a) Association between UUER (mg/24 h) and urine volume (mL/24 h) in univariate model. (b) Association between UUER (mg/24 h) and serum copeptin concentration (pmol/L) in univariate model. (c) Association between UUER (mg/24 h) and serum copeptin concentration (pmol/L) according to tertiles of urine volume (mL/24 h) in partially adjusted model. UUER is square root transformed. Serum copeptin and urine volume are log transformed. All models are adjusted for center as a fixed effect and family as a random effect. UUER, urine uromodulin excretion rate.

**FIGURE 2:**
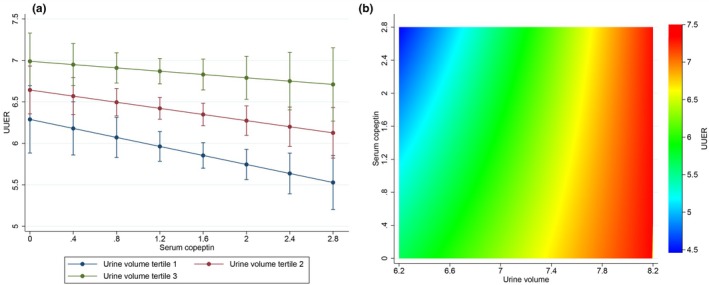
Association between UUER (mg/24 h) and serum copeptin concentration (pmol/L) according to urine volume (mL/24 h) in the fully adjusted model (*N* = 937). (a) Urine volume expressed as tertiles of distribution. (b) Urine volume expressed as a continuous variable. Model is adjusted for: Age, gender, diabetes, eGFR, kidney volume, and diuretics. Model is also adjusted for center as a fixed effect and family as a random effect. UUER is square root transformed. Serum copeptin and urine volume are log transformed. eGFR, estimated glomerular filtration rate; UUER, urine uromodulin excretion rate.

The unadjusted association of UUER with urine volume as well as with serum copeptin was analyzed in two distinct univariate models. UUER was positively associated with urine volume (*β* = 1.24, 95% CI 1.01–1.47, *p* < 0.001) (Figure 1a) and negatively associated with serum copeptin concentration (*β* = −0.37, 95% CI −0.53 to −0.20, *p* < 0.001) (Figure 1b). An alternative representation is provided in Figure S3a,b representing individual observations. In order to test whether copeptin may directly act on UUER independently of variations in urine volume, we analyzed the combined association of UUER with both urine volume and serum copeptin concentration in a single partially adjusted model. UUER was positively associated with urine volume (*β* = 0.83, 95% CI 0.24–1.42, *p* = 0.006) but not associated with serum copeptin concentration (*β* = −2.05, 95% CI −4.90 to 0.80, *p* = 0.159) (Figure 1c). Serum copeptin did not modify the association between UUER and urine volume as indicated by the non‐significant interaction term (*p* = 0.174). In order to account for other potential confounders, we analyzed the combined association of UUER with every a priori specified covariate including urine volume and serum copeptin concentration in a single fully adjusted model. We confirmed that UUER was positively associated with urine volume (*β* = 0.84, 95% CI 0.27–1.41, *p* = 0.004) but not associated with serum copeptin concentration (*β* = −1.71, 95% CI −4.46 to 1.04, *p* = 0.224). The association between UUER and serum copeptin concentration according to tertiles of urine volume in this fully adjusted model is depicted in Figure 2a. An alternative description with urine volume also represented as a continuous variable is provided in Figure 2b. Serum copeptin did not modify the association between UUER and urine volume as indicated by the non‐significant interaction term (*p* = 0.252). In this fully adjusted model, UUER was negatively associated with the presence of diabetes and positively associated with eGFR as well as kidney volume, whereas no influence of age, gender, or the use of diuretics was detected (Table 2). Variance inflation factor showed no collinearity between UUER, urine volume and serum copeptin concentration in the fully adjusted model. Model diagnostics for the fully adjusted model supported adequate model fit, with approximately normal residuals and no significant heteroscedasticity.

These data, based on successive models with increasing adjustment for potential confounders, indicate that UUER is mainly determined by urine volume independently of serum copeptin.

### 3.3 Determinants of urine concentration

We next tested the potential association between UUER and urine concentration as indicated by urine osmolality while accounting for serum copeptin and urine volume (Table 3).

**TABLE 3:** Determinants of urine osmolality^a^ (mOsm/kg) in univariate, partially adjusted as well as fully adjusted models (*N* = 937).

	Univariate models		Partially adjusted model^b^		Fully adjusted model^b^		
	*β* coefficient	*p* Value	*β* coefficient	*p* Value	*β* coefficient	95% CI	*p* Value
UUER^a^ (mg/24 h)	−0.23	**0.016**	0.18	0.374	0.47	0.20 to 0.75	**0.001**
Serum copeptin^c^ (pmol/L)	3.99	**<0.001**	4.95	**<0.001**	1.66	0.46 to 2.85	**0.006**
Urine volume^c^ (mL/24 h)	−8.21	**<0.001**			−7.99	−8.45 to −7.52	**<0.001**
Age (per 10 years)	−0.20	**0.011**			−0.07	−0.22 to 0.07	0.309
Gender (women)	−3.19	**<0.001**			−1.83	−2.23 to −1.44	**<0.001**
Diabetes (yes)	1.01	0.148			−0.16	−0.98 to 0.64	0.684
eGFR (per 10 mL/min/1.73m^2^)	0.27	**0.001**			0.11	−0.03 to 0.25	0.136
Kidney volume (per 100 mL)	2.80	**<0.001**			1.22	0.70 to 1.75	**<0.001**
Diuretics (yes)	0.88	0.171			−0.31	−1.07 to 0.43	0.411

*Note*: All models are adjusted for center as a fixed effect and family as a random effect (not shown). Bold values correspond to *p* value < 0.05.

Abbreviations: eGFR, estimated glomerular filtration rate; UUER, urine uromodulin excretion rate.

^a^ Square root transformed.

^b^ Interaction between UUER and serum copeptin is considered (not shown, see text).

^c^ Log transformed.

**FIGURE 3:**
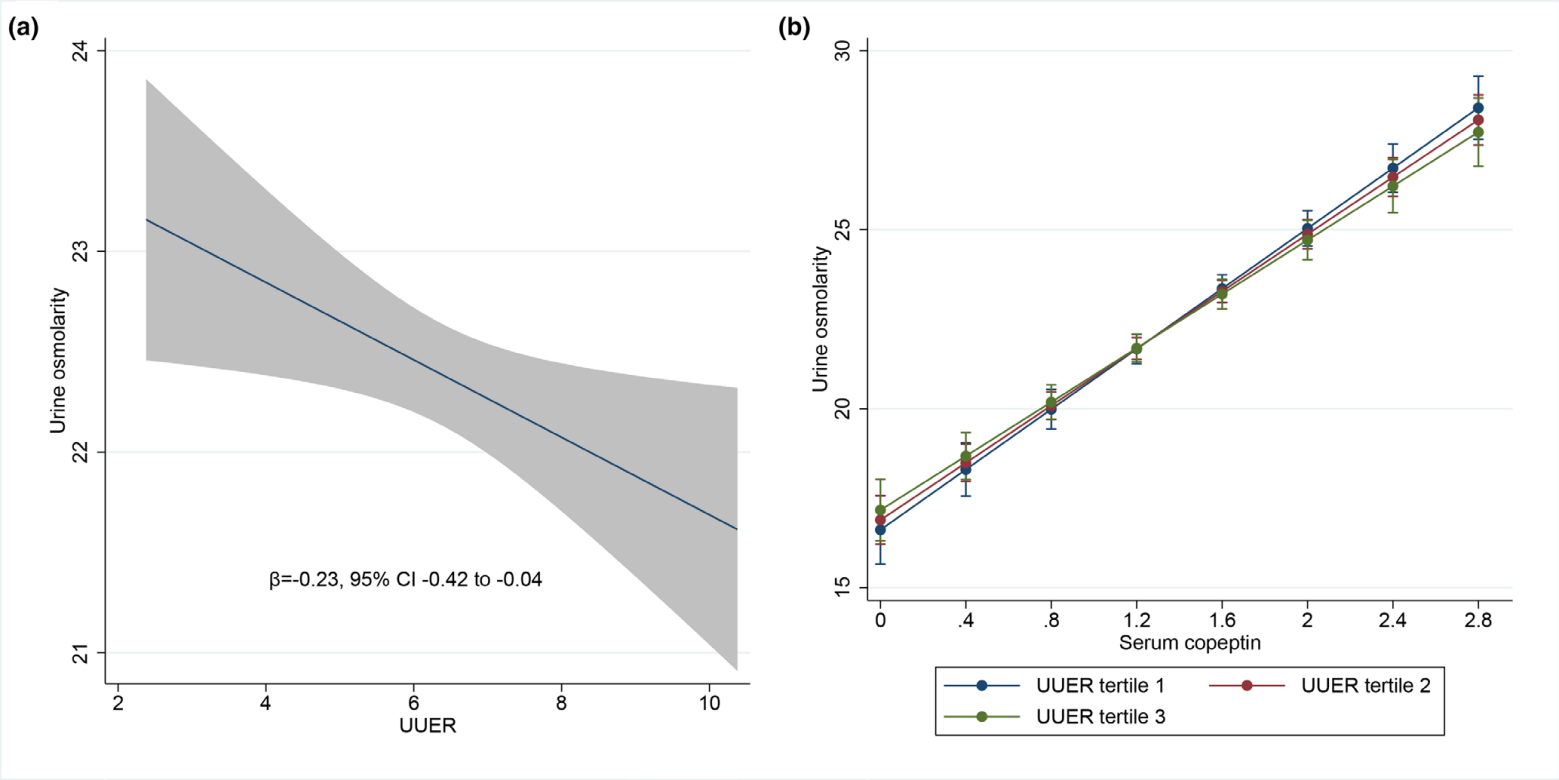
Determinants of urine osmolality (mOsm/kg) in univariate model and single partially adjusted model (*N* = 937). (a) Association between urine osmolality (mOsm/kg) and UUER (mg/24 h) in univariate model. (b) Association between urine osmolality (mOsm/kg) and serum copeptin concentration (pmol/L) according to tertiles of UUER (mg/24 h) in partially adjusted model. Urine osmolality and UUER are square root transformed. Serum copeptin concentration is log transformed. All models are adjusted for center as a fixed effect and family as a random effect. UUER, urine uromodulin excretion rate.

**FIGURE 4:**
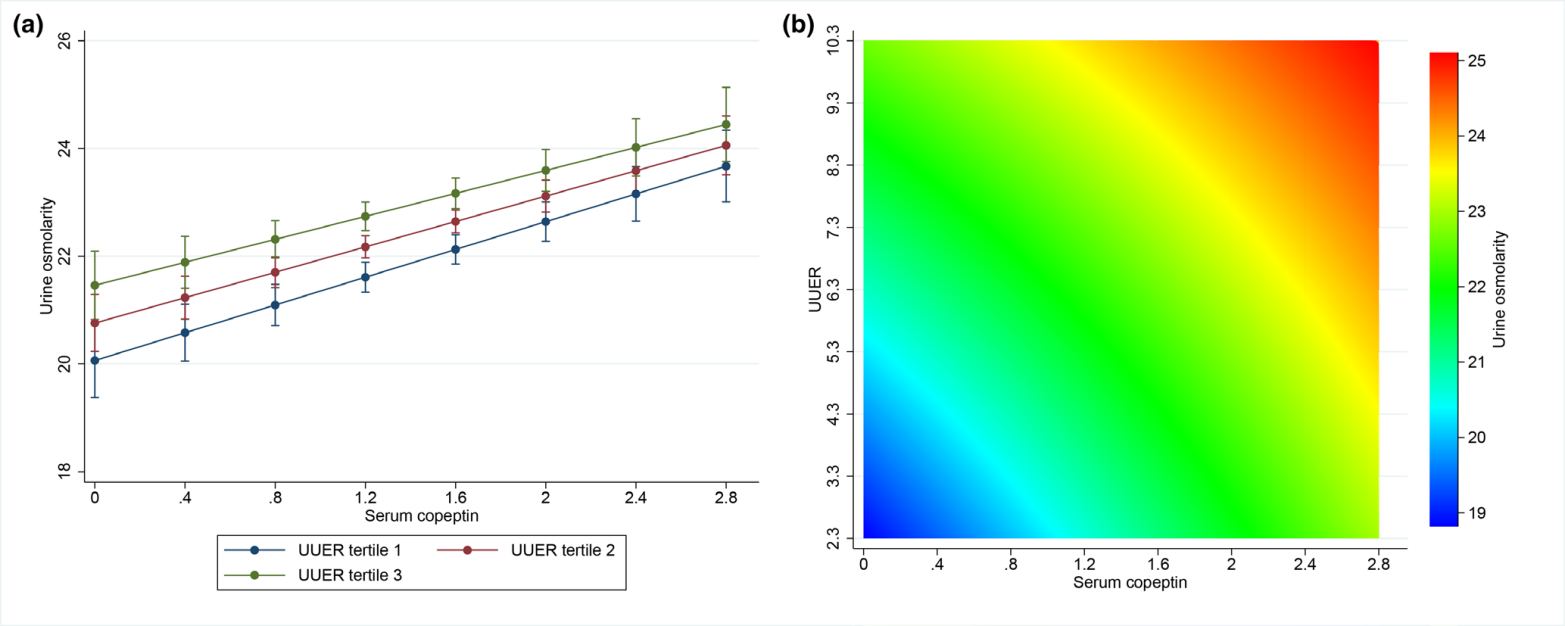
Association between urine osmolality (mOsm/kg) and serum copeptin concentration (pmol/L) according to UUER (mg/24 h) in the fully adjusted model (*N* = 937). (a) UUER expressed as tertiles of distribution. (b) UUER expressed as a continuous variable. Model is adjusted for: Urine volume, age, gender, diabetes, eGFR, kidney volume, and diuretics. Model is also adjusted for center as a fixed effect and family as a random effect. Urine osmolality and UUER are square root transformed. Serum copeptin concentration is log transformed. eGFR, estimated glomerular filtration rate; UUER, urine uromodulin excretion rate.

The unadjusted association of urine osmolality with UUER was tested in a univariate model. Urine osmolality was negatively associated with UUER (β = −0.23, 95% CI −0.42 to −0.04, *p* = 0.016) (Figure 3a). An alternative representation is provided in Figure S4 representing individual observations in order to account for the effect of copeptin on urine osmolality, we analyzed the combined association of urine osmolality with both UUER and serum copeptin concentration in a single partially adjusted model. Urine osmolality was positively associated with serum copeptin concentration (*β* = 4.95, 95% CI 3.16–6.73, *p* < 0.001) but not associated with UUER (*β* = 0.18, 95% CI −0.22 to 0.60, *p* = 0.374) (Figure 3b). Serum copeptin did not modify the association between urine osmolality and UUER as indicated by the non‐significant interaction term (*p* = 0.274). In order to account for the effect of urine volume on UUER (see above), we analyzed the combined association of urine osmolality with every a priori specified covariate including UUER, serum copeptin concentration and urine volume in a single fully adjusted model. As expected, urine osmolality was positively associated with serum copeptin concentration (*β* = 1.66, 95% CI 0.46–2.85, *p* = 0.006) and negatively associated with urine volume (*β* = −7.99, 95% CI −8.45 to −7.52, *p* < 0.001). Importantly, urine osmolality was positively associated with UUER (*β* = 0.47, 95% CI 0.20–0.75, *p* = 0.001). The association between urine osmolality and serum copeptin concentration according to tertiles of UUER in this fully adjusted model is depicted in Figure 4a. An alternative description with UUER also represented as a continuous variable is provided in Figure 4b. Serum copeptin did not modify the association between urine osmolality and UUER as indicated by the nonsignificant interaction term (*p* = 0.400). In this fully adjusted model, urine osmolality was positively associated with male gender as well as kidney volume and not associated with age, diabetes, eGFR, or the use of diuretics. Variance inflation factor showed no collinearity between urine osmolality, UUER, serum copeptin concentration and urine volume in the fully adjusted model. Model diagnostics for the fully adjusted model supported adequate model fit, with approximately normal residuals and no significant heteroscedasticity.

Together, these results indicate that urine osmolality is additively determined by serum copeptin as well as UUER once accounting for the effect of urine volume.

### 3.4 Sensitivity analyses

Analyses were repeated with further adjustment for urine creatinine excretion (mg/kg/24 h). Main results were similar and UUER remained positively associated with urine volume (Table S1), while urine osmolality remained positively associated with UUER (Table S2). Analyses were repeated with further adjustment for urine sodium excretion (mmol/24 h). Main results were similar and UUER remained positively associated with urine volume (Table S3).

Analyses were repeated excluding participants with CKD as defined by eGFR <60 mL/min/1.73m^2^. Consequently, 27 participants were excluded and 910 were included. Main results were similar and UUER remained positively associated with urine volume (Table S4), while urine osmolality remained positively associated with UUER (Table S5).

The main results were also similar when using calculated urine osmolality instead of measured urine osmolality and urine osmolality remained positively associated with UUER (Table S6).

Finally, analyses were repeated including 90 participants a priori considered outliers as specified in the methods section, and 1′027 participants were thus analyzed (Figure S1). Main results were similar and UUER remained positively associated with urine volume (Table S7), while urine osmolality remained positively associated with UUER (Table S8).

## 4 DISCUSSION

**FIGURE 5:**
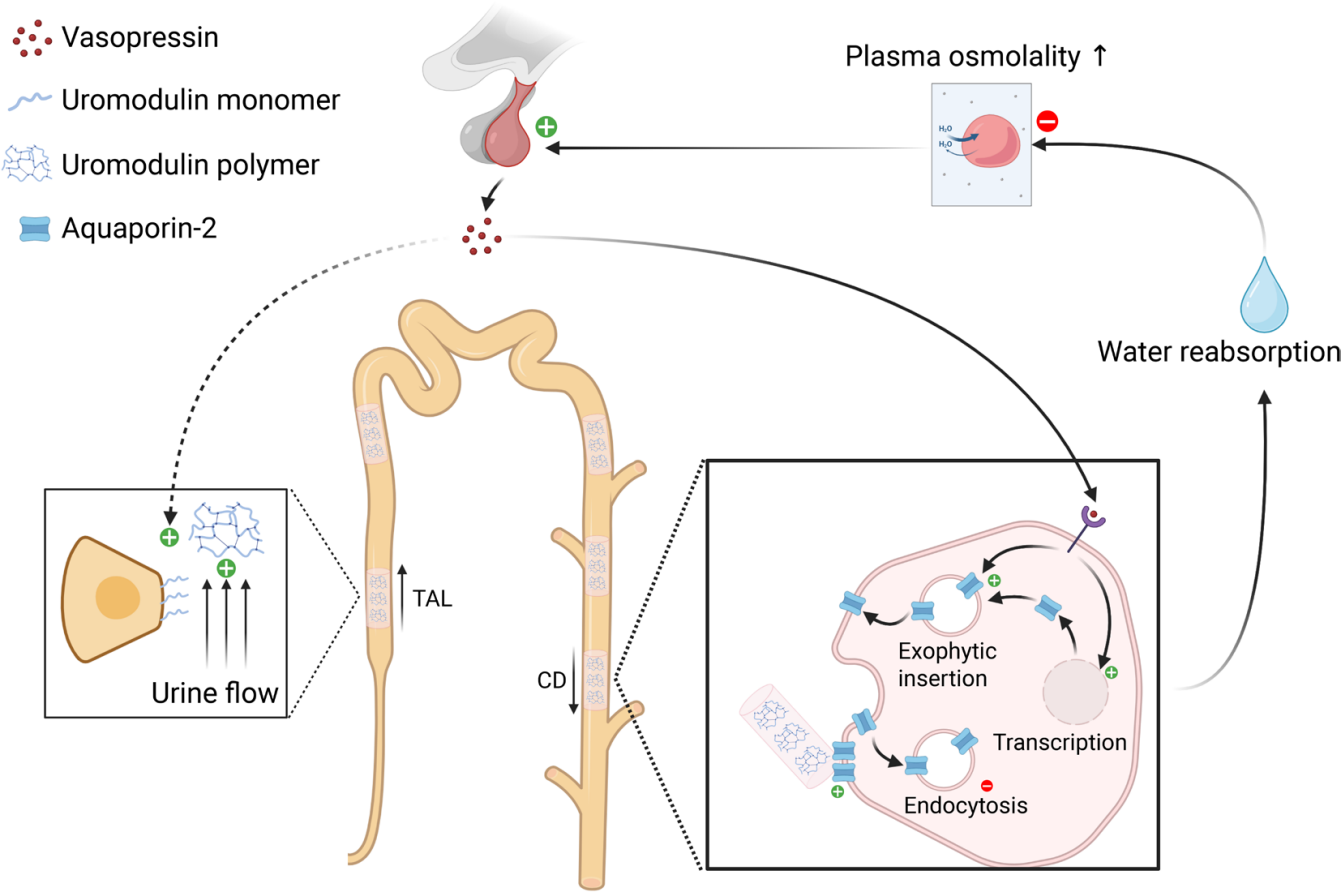
Physiological regulation of uromodulin excretion and potential role in water handling. In the thick ascending limb (TAL), an increase in urine flow promotes the release of uromodulin monomers from the apical plasma membrane into the urine. Animal models have suggested that vasopressin may also increase uromodulin secretion by acting on TAL cells (dashed arrow). Uromodulin monomers aggregate into polymers forming the matrix component of hyaline casts. In the collecting duct (CD), uromodulin polymers interact from the lumen with the aquaporin‐2 (AQP2) water channels inserted in the apical plasma membrane. This interaction decreases the rate of internalization of AQP2, thus facilitating water reabsorption in addition to the established effects of vasopressin on the transcription and activation of AQP2 in these cells. AQP2, aquaporin 2; CD, collecting duct; TAL, thick ascending limb of Henle. Figure designed with Biorender.

In this study, we tested in a general healthy population the interplay between water balance, uromodulin excretion and the ability to concentrate urine. First, we show that the uromodulin excretion rate is mainly associated with urine volume, without a significant relationship to serum copeptin concentration. Second, we show that urine osmolality is additively associated with both serum copeptin concentration and uromodulin excretion. These results provide new insights into the physiological regulation of uromodulin excretion and its potential role in water handling by the distal nephron (Figure 5).

### 4.1 Determinants of urine uromodulin excretion

Previous population studies established that the UUER is driven by common genetic variants in the *UMOD‐PDILT* locus, the level of kidney function (as monitored by eGFR), the fractional excretion of sodium, and the length/volume of the kidney (Pruijm et al., 2016; Troyanov et al., 2016). Since uromodulin is essentially produced and released from the TAL cells, which are critical for the urinary concentrating ability, a potential interaction between water balance, vasopressin, and uromodulin excretion has been raised. In healthy individuals, UUER is positively associated with urine volume and increases with acute water loading and increased urine flow rate (LaFavers et al., 2023; Lynn et al., 1982; Pruijm et al., 2016). These observations suggest that the release of uromodulin from the apical plasma membrane may be regulated by the luminal flow, which could increase the level of proteolytic cleavage by the hepsin serine protease (Brunati et al., 2015). Of note, uromodulin has been shown to be localized on the primary cilium of the TAL cells, an observation that could support the link between flow‐sensing and uromodulin release (Zaucke et al., 2010).

The antidiuretic effect of vasopressin is mediated by type 2 vasopressin receptor (V2R), which increases intracellular cAMP through activating the Gs‐pathway and stimulating protein kinase A (PKA) and other kinases (Zaucke et al., 2010). The V2R is expressed in the basolateral membrane of cells lining the cortical and medullary TAL, DCT, and CD (Mutig et al., 2007). The AVP‐V2R system in the TAL participates in the regulation of renal water handling by modulating the activity of NKCC2 and thus the generation of the interstitial osmolality (Bachmann & Mutig, 2017). Thus far, the direct effect of AVP on UUER has not been tested in humans. Acute administration of the V2R agonist desmopressin in mice markedly increased the UUER, without affecting the production or degradation of uromodulin in the kidney (Nanamatsu et al., 2021). Conversely, chronic desmopressin infusion (for 1–3 weeks) in Brattleboro rats, which have central diabetes insipidus, significantly lowered UUER (Bachmann et al., 1991). Differences between short‐ and longer‐term effects of desmopressin in the rodent models may potentially reflect a depletion in the uromodulin stock in TAL cells occurring with chronic administration of the hormone, similar to what is observed in desmopressin‐induced von Willebrand factor endothelial secretion (Mannucci et al., 1992).

In the present study, we characterized clinical determinants of UUER accounting both for urine volume as well as serum copeptin at steady state in a healthy general population. Simple univariate associations indicated that UUER increases linearly with urine volume and decreases with serum copeptin concentration. Conceptually, the univariate relationship between serum copeptin and UUER could thus be indirectly mediated by variations of urine volume or by a direct effect of vasopressin on UUER, as reported in animal models. Using multivariate models accounting for potential confounders as well as interacting effects between key variables, we found that urine volume remained strongly associated with UUER, while the relationship with serum copeptin became nonsignificant. These findings confirm the independent association between UUER and urine volume, with no apparent direct or additional effect of vasopressin on UUER. Taken together, our findings do not support a significant direct stimulatory effect of vasopressin on UUER in humans at steady state. On the contrary, the tendency toward a negative (though nonsignificant) association between serum copeptin and UUER, with borderline *p* values in some secondary models, is compatible with a possible depletory effect of chronic vasopressin activity on kidney uromodulin abundance, as suggested by experimental studies (LaFavers et al., 2023; Nanamatsu et al., 2021).

### 4.2 Effect of uromodulin excretion on urine concentration

Recent studies indicate that, once released in the lumen by the TAL cells, uromodulin filaments may affect the activity of transporters localized in cells lining downstream tubular segments (Schaeffer et al., 2021). These effects may include enhanced phosphorylation and apical trapping of AQP2 channels in the CD, which could promote water reabsorption under the influence of vasopressin (Takata et al., 2022). To test the clinical relevance of this experimental hypothesis, we constructed several models to characterize the potential physiological role of urine uromodulin in water reabsorption. Using simple univariate analysis, higher UUER was associated with lower urine osmolality, and thus lower water reabsorption. However, key determinants of urine concentration, such as vasopressin activity and urine volume, were not accounted for in this initial model. Accounting for serum copeptin concentration alone rendered the relationship between UUER and urine osmolality non‐significant. However, as we showed that UUER is highly dependent on urine volume, only by accounting for the independent effect of urine volume did it become apparent that UUER was in fact associated with urine osmolality, with higher UUER corresponding to increased urine osmolality. In other terms, for any given value of serum copeptin and urine volume, a higher UUER would correspond to a higher urine osmolality. These results support the hypothesis of an additive effect of uromodulin on distal water reabsorption, which could be mediated by its interaction with AQP2 in CD cells (Takata et al., 2022). The lack of an interacting effect between serum copeptin and UUER in the prediction of urine osmolality suggests an additive effect of vasopressin and uromodulin on water reabsorption.

Strengths of our study comprise its population‐based design and its large sample size of healthy participants allowing it to clinically inform experimental hypotheses. A major asset is the simultaneous and precise measurement of serum copeptin, urine uromodulin as well as urine parameters based on 24 h collections, allowing a comprehensive description of water balance in healthy subjects. We also relied on refined statistical models integrating many potential confounding variables as well as robust sensitivity analyses.

The main limitation of our study is inherent to its observational design. More specifically, associations highlighted in this paper, while robust and highly significant, do not necessarily imply a causative relationship between considered variables. However, we believe this inherent limitation should be mitigated as our study was designed to clinically inform physiological hypotheses formulated a priori in animal studies. Our findings should thus be perceived as clinical evaluations of experimental hypotheses. Other secondary limitations comprise a strictly Caucasian population and the lack of a validation cohort. However, we are not aware of another healthy population cohort with a comprehensive characterization of water balance in relation to uromodulin excretion.

In conclusion, we designed an observational study to clinically inform physiological hypotheses arising from animal studies regarding the regulation and potential effect of uromodulin excretion on water reabsorption. Our results indicate that urine uromodulin excretion strongly associates with urine volume and influences urine osmolality in addition to vasopressin/copeptin levels. These data substantiate the role of urine flow in regulating uromodulin excretion and suggest an additive effect of uromodulin on urine concentration, alongside vasopressin.

## AUTHOR CONTRIBUTIONS

**David A. Jaques:** Formal analysis; funding acquisition; investigation; methodology; software; visualization. **Théodore Pasquier:** Formal analysis; visualization. **Menno Pruijm:** Data curation; funding acquisition; investigation; supervision. **Daniel Ackermann:** Funding acquisition; project administration; supervision. **Murielle Bochud:** Funding acquisition; investigation; methodology; supervision. **Olivier Devuyst:** Conceptualization; data curation; funding acquisition; investigation; methodology; project administration; resources; validation. **Belen Ponte:** Conceptualization; formal analysis; funding acquisition; investigation; methodology; project administration.

## FUNDING INFORMATION

OD is supported by the European Union's Horizon 2020 Research and Innovation Program under Marie Skłodowska‐Curie Grant 860977, European Reference Network for Rare Kidney Diseases Project 739532, the Swiss National Science Foundation Grant 310030‐189044, and the University Research Priority Program Innovative Therapies in Rare Diseases (ITINERARE) at the University of Zurich. SKIPOGH was supported by a grant from the Swiss National Science Foundation (FN 33CM30‐124087). Copeptin dosage was supported by local funding from the Geneva University Hospitals (PRD 10‐2013‐I).

## CONFLICT OF INTEREST STATEMENT

Authors declare no conflict of interest.

## Supporting information


Appendix S1.

## Data Availability

The data underlying this article will be shared on reasonable request to the corresponding author.
