# Supplementary material for: Interplay between uromodulin excretion and water balance in the general adult population
Source: Physiol Rep. 2026 Mar 29;14(7):e70844. doi: 10.14814/phy2.70844 (PMC13140577; doi:10.14814/phy2.70844)
Supplement: Supplementary file 1 — Appendix S1. [file PHY2-14-e70844-s001.docx]

**Interplay between Uromodulin Excretion and Water Balance in the General Population**

**Supplementary Material**

**Supplemental table S1**: Determinants of UUER^a^ (mg/24h) in fully adjusted model, with further adjustment for urine creatinine excretion (mg/kg/24h) (N=931).

|  | **β coefficient** | **95% CI** | **p value** |
| --- | --- | --- | --- |
| Urine volume^b^ (mL/24h) | 0.65 | 0.09 to 1.22 | **0.023** |
| Serum copeptin^b^ (pmol/L) | -2.13 | -4.85 to 0.57 | 0.123 |
| Age (per 10 years) | 0.03 | -0.05 to 0.11 | 0.442 |
| Gender (women) | 0.08 | -0.14 to 0.32 | 0.449 |
| Diabetes (yes) | -0.74 | -1.17 to -0.32 | **<0.001** |
| eGFR (per mL/min/1.73m^2^) | 0.09 | 0.01 to 0.17 | **0.020** |
| Kidney volume (per 100 mL) | 0.53 | 0.25 to 0.81 | **<0.001** |
| Diuretics (yes) | 0.27 | -0.11 to 0.66 | 0.166 |
| Urine creatinine excretion^a^ (mg/kg/24h) | 0.58 | 0.37 to 0.79 | **<0.001** |

*Abbreviations: UUER, urinary uromodulin excretion rate; eGFR, estimated glomerular filtration rate.*

a: Square root transformed.

b: Log transformed.

Model is adjusted for center as a fixed effect and family as a random effect (not shown). Interaction between urine volume and serum copeptin centration is considered (not shown).

**Supplemental table S2**: Determinants of urine osmolality^a^ (mOsm/kg) in fully adjusted model, with further adjustment for urine creatinine excretion (mg/kg/24h) (N=931).

|  | **β coefficient** | **95% CI** | **p value** |
| --- | --- | --- | --- |
| UUER^a^ (mg/24h) | 0.41 | 0.16 to 0.67 | **0.001** |
| Serum copeptin^b^ (pmol/L) | 1.81 | 0.71 to 2.91 | **0.001** |
| Urine volume^b^ (mL/24h) | -8.26 | -8.69 to -7.83 | **<0.001** |
| Age (per 10 years) | 0.26 | 0.11 to 0.40 | **<0.001** |
| Gender (women) | -0.66 | -1.07 to -0.25 | **0.001** |
| Diabetes (yes) | 0.01 | -0.73 to 0.76 | 0.972 |
| eGFR (per 10 mL/min/1.73m^2^) | 0.13 | -0.00 to 0.27 | 0.054 |
| Kidney volume (per 100 mL) | 1.67 | 1.17 to 2.16 | **<0.001** |
| Diuretics (yes) | -0.14 | -0.84 to 0.54 | 0.674 |
| Urine creatinine excretion^a^ (mg/kg/24h) | 2.40 | 2.02 to 2.77 | **<0.001** |

*Abbreviations: UUER, urinary uromodulin excretion rate; eGFR, estimated glomerular filtration rate.*

a: Square root transformed.

b: Log transformed.

Model is adjusted for center as a fixed effect and family as a random effect (not shown).

Interaction between UUER and serum copeptin is considered (not shown).

**Supplemental Table S3:** Determinants of UUER (mg/24h) in fully adjusted model, with further adjustment for urine sodium excretion (mmol/24h) (N=937).

|  | **Fully adjusted model^b^** | | |
| --- | --- | --- | --- |
|  | **β coefficient** | **95% CI** | **p value** |
| Urine volume^c^ (mL/24h) | 0.64 | 0.08 to 1.21 | **0.025** |
| Serum copeptin^c^ (pmol/L) | -1.39 | -4.04 to 1.36 | 0.332 |
| Age (per 10 years) | -0.05 | -0.12 to 0.02 | 0.205 |
| Gender (women) | -0.08 | -0.29 to 0.12 | 0.425 |
| Diabetes (yes) | -0.78 | -1.20 to -0.36 | **<0.001** |
| eGFR (per 10 mL/min/1.73m^2^) | 0.07 | -0.00 to 0.14 | 0.073 |
| Kidney volume (per 100 mL) | 0.32 | 0.04 to 0.61 | **0.023** |
| Diuretics (yes) | 0.24 | -0.13 to 0.63 | 0.209 |
| Urinary sodium excretion (mmol24h) | 0.12 | 0.08 to 0.16 | <0.001 |

**Supplemental table S4**: Determinants of UUER^a^ (mg/24h) in fully adjusted model, excluding participants with CKD (N=910).

|  | **β coefficient** | **95% CI** | **p value** |
| --- | --- | --- | --- |
| Urine volume^b^ (mL/24h) | 0.72 | 0.15 to 1.30 | **0.013** |
| Serum copeptin^b^ (pmol/L) | -2.52 | -5.31 to 0.26 | 0.076 |
| Age (per 10 years) | -0.08 | -0.16 to -0.00 | **0.040** |
| Gender (women) | -0.17 | -0.38 to 0.03 | 0.102 |
| Diabetes (yes) | -0.86 | -1.29 to -0.43 | **<0.001** |
| eGFR (per 10 mL/min/1.73m^2^) | 0.02 | -0.05 to 0.11 | 0.501 |
| Kidney volume (per 100 mL) | 0.49 | 0.20 to 0.78 | **0.001** |
| Diuretics | 0.39 | -0.02 to 0.81 | 0.065 |

*Abbreviations: UUER, urinary uromodulin excretion rate; eGFR, estimated glomerular filtration rate.*

a: Square root transformed.

b: Log transformed.

Model is adjusted for center as a fixed effect and family as a random effect (not shown).

Interaction between serum copeptin centration and urine volume is considered (not shown).

**Supplemental table S5**: Determinants of urine osmolality^a^ (mOsm/kg) in fully adjusted model, excluding participants with CKD (N=910).

|  | **β coefficient** | **95% CI** | **p value** |
| --- | --- | --- | --- |
| UUER^a^ (mg/24h) | 0.42 | 0.14 to 0.70 | **0.003** |
| Serum copeptin^b^ (pmol/L) | 1.46 | 0.23 to 2.70 | **0.020** |
| Urine volume^b^ (mL/24h) | -7.98 | -8.45 to -7.50 | **<0.001** |
| Age (per 10 years) | -0.09 | -0.25 to 0.05 | 0.204 |
| Gender (women) | -1.78 | -2.18 to -1.37 | **<0.001** |
| Diabetes (yes) | -0.21 | -1.04 to 0.61 | 0.616 |
| eGFR (per 10 mL/min/1.73m^2^) | 0.05 | -0.10 to 0.21 | 0.497 |
| Kidney volume (per 100 mL) | 1.36 | 0.81 to 1.90 | **<0.001** |
| Diuretics | -0.55 | -1.36 to 0.25 | 0.179 |

*Abbreviations: UUER, urinary uromodulin excretion rate; eGFR, estimated glomerular filtration rate.*

a: Square root transformed.

b: Log transformed.

Model is adjusted for center as a fixed effect and family as a random effect (not shown).

Interaction between UUER and serum copeptin is considered (not shown).

**Supplemental table S6**: Determinants of urine osmolality^a^ (mOsm/kg) in fully adjusted model, using calculated urine osmolality (N=929).

|  | **β coefficient** | **95% CI** | **p value** |
| --- | --- | --- | --- |
| UUER^a^ (mg/24h) | 0.29 | 0.02 to 0.56 | **0.029** |
| Serum copeptin^b^ (pmol/L) | 0.85 | -0.31 to 2.01 | 0.152 |
| Urine volume^b^ (mL/24h) | -7.71 | -8.16 to -7.27 | **<0.001** |
| Age (per 10 years) | -0.07 | -0.22 to 0.06 | 0.285 |
| Gender (women) | -1.78 | -2.17 to -1.39 | **<0.001** |
| Diabetes (yes) | -0.50 | -1.31 to 0.30 | 0.219 |
| eGFR (per 10 mL/min/1.73m^2^) | 0.10 | -0.03 to 0.25 | 0.131 |
| Kidney volume (per 100mL) | 1.41 | 0.91 to 1.92 | **<0.001** |
| Diuretics (yes) | -0.03 | -0.77 to 0.69 | 0.919 |

*Abbreviations: UUER, urinary uromodulin excretion rate; eGFR, estimated glomerular filtration rate.*

a: Square root transformed.

b: Log transformed.

Model is adjusted for center as a fixed effect and family as a random effect (not shown).

Interaction between UUER and serum copeptin is considered (not shown).

**Supplemental table S7**: Determinants of UUER^a^ (mg/24h) in fully adjusted model, including outliers (N=1’027).

|  | **β coefficient** | **95% CI** | **p value** |
| --- | --- | --- | --- |
| Urine volume^b^ (mL/24h) | 0.78 | 0.26 to 1.30 | **0.003** |
| Serum copeptin^b^ (pmol/L) | -2.34 | -4.76 to 0.07 | 0.058 |
| Age (per 10 years) | -0.03 | -0.11 to 0.04 | 0.349 |
| Gender (women) | -0.18 | -0.40 to 0.03 | 0.092 |
| Diabetes (yes) | -0.92 | -1.36 to -0.49 | **<0.001** |
| eGFR (per 10 mL/min/1.73m^2^) | 0.14 | 0.06 to 0.22 | **<0.001** |
| Kidney volume (per 100 mL) | 0.54 | 0.26 to 0.82 | **<0.001** |
| Diuretics | 0.32 | -0.05 to 0.27 | 0.121 |

*Abbreviations: UUER, urinary uromodulin excretion rate; eGFR, estimated glomerular filtration rate.*

a: Square root transformed.

b: Log transformed.

Model is adjusted for center as a fixed effect and family as a random effect (not shown).

Interaction between serum copeptin centration and urine volume is considered (not shown).

**Supplemental table S8**: Determinants of urine osmolality^a^ (mOsm/kg) in fully adjusted model, including outliers (N=1’027).

|  | **β coefficient** | **95% CI** | **p value** |
| --- | --- | --- | --- |
| UUER^a^ (mg/24h) | 0.69 | 0.45 to 0.94 | **<0.001** |
| Serum copeptin^b^ (pmol/L) | 2.39 | 1.39 to 3.39 | **<0.001** |
| Urine volume^b^ (mL/24h) | -7.88 | -8.30 to -7.46 | **<0.001** |
| Age (per 10 years) | -0.12 | -0.27 to 0.01 | 0.079 |
| Gender (women) | -1.87 | -2.27 to -1.47 | **<0.001** |
| Diabetes (yes) | -0.12 | -0.92 to 0.68 | 0.766 |
| eGFR (per 10 mL/min/1.73m^2^) | 0.09 | -0.05 to 0.23 | 0.212 |
| Kidney volume (per 100 mL) | 1.22 | 0.72 to 1.73 | **<0.001** |
| Diuretics | -0.45 | -1.20 to 0.29 | 0.237 |

*Abbreviations: UUER, urinary uromodulin excretion rate; eGFR, estimated glomerular filtration rate.*

a: Square root transformed.

b: Log transformed.

Model is adjusted for center as a fixed effect and family as a random effect (not shown).

Interaction between UUER and serum copeptin is considered (not shown).

**Supplemental Figure S1**: Study flowchart.


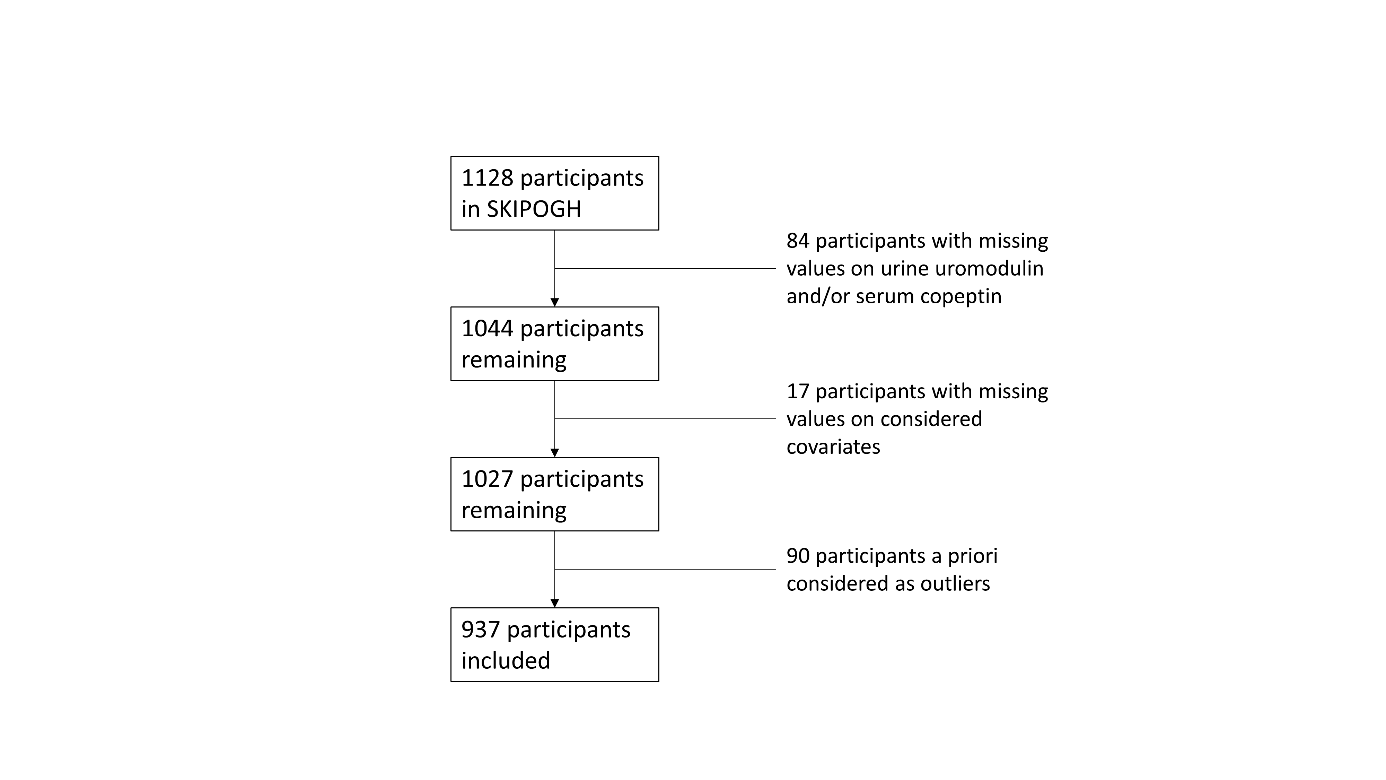


**Supplemental Figure S2a:** Association between plasma osmolality (mOsm/kg) and serum copeptin (pmol/L) in univariate model.

Serum copeptin is log transformed.

**Supplemental Figure S2b**: Association between serum copeptin (pmol/L) and urine osmolality (mOsm/kg) in univariate model.

Serum copeptin is log transformed. Urine osmolality is square root transformed.

**Supplemental Figure S2c**: Association between serum copeptin (pmol/L) and urine volume (mL/24h) in univariate model.

Serum copeptin and urine volume are log transformed.


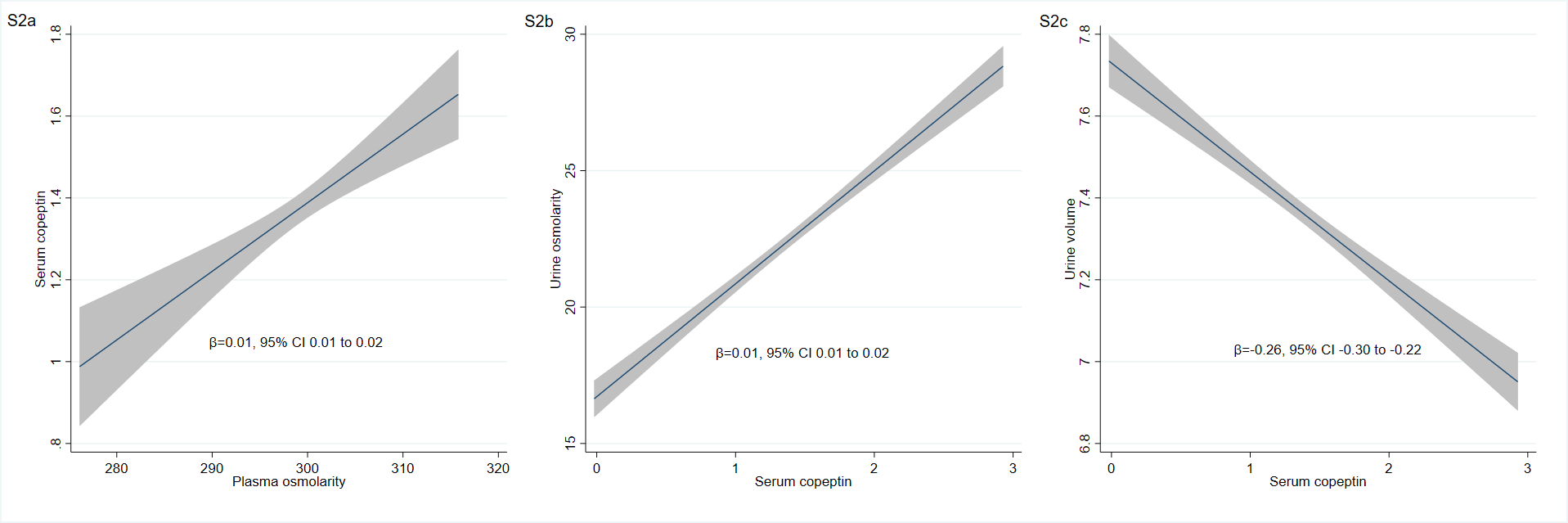


**Supplemental Figure S3a**: Association between UUER (mg/24h) and urine volume (mL/24h) in univariate model.

**Supplemental S3b**: Association between UUER (mg/24h) and serum copeptin concentration (pmol/L) in univariate model.

UUER is square root transformed. Serum copeptin and urine volume are log transformed.

All models are adjusted for center as a fixed effect and family as a random effect.


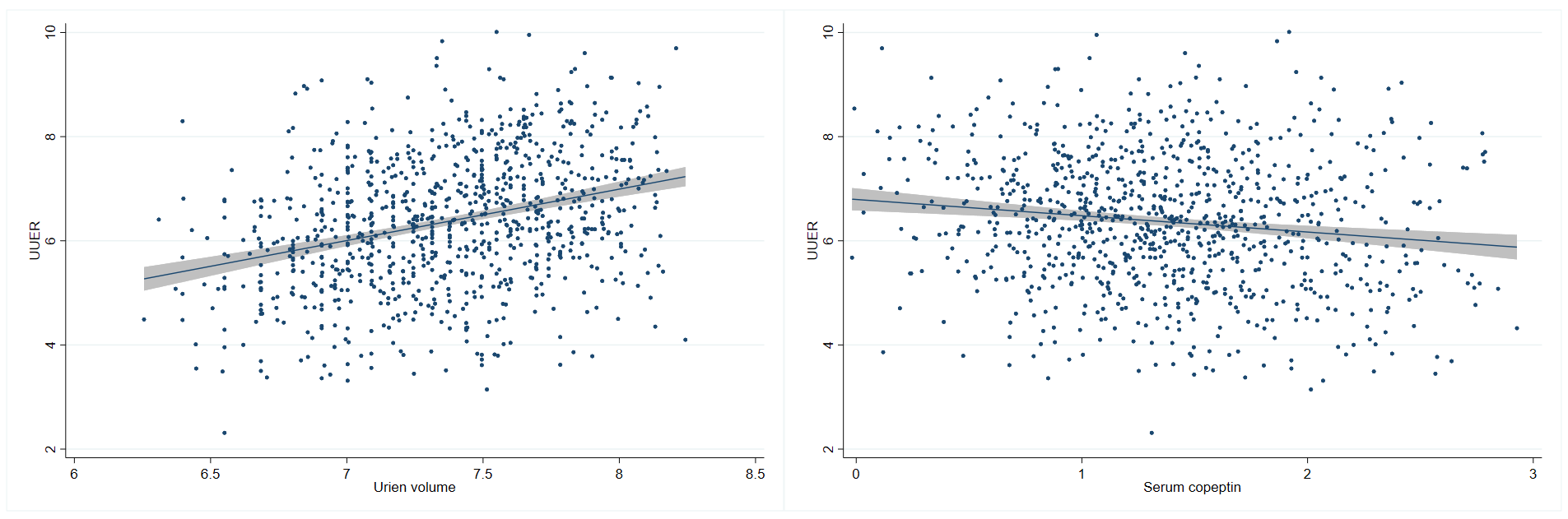


**Supplemental Figure S4**: Association between urine osmolality (mOsm/kg) and UUER (mg/24h) in univariate model.

Urine osmolality and UUER are square root transformed. Serum copeptin concentration is log transformed.

All models are adjusted for center as a fixed effect and family as a random effect.


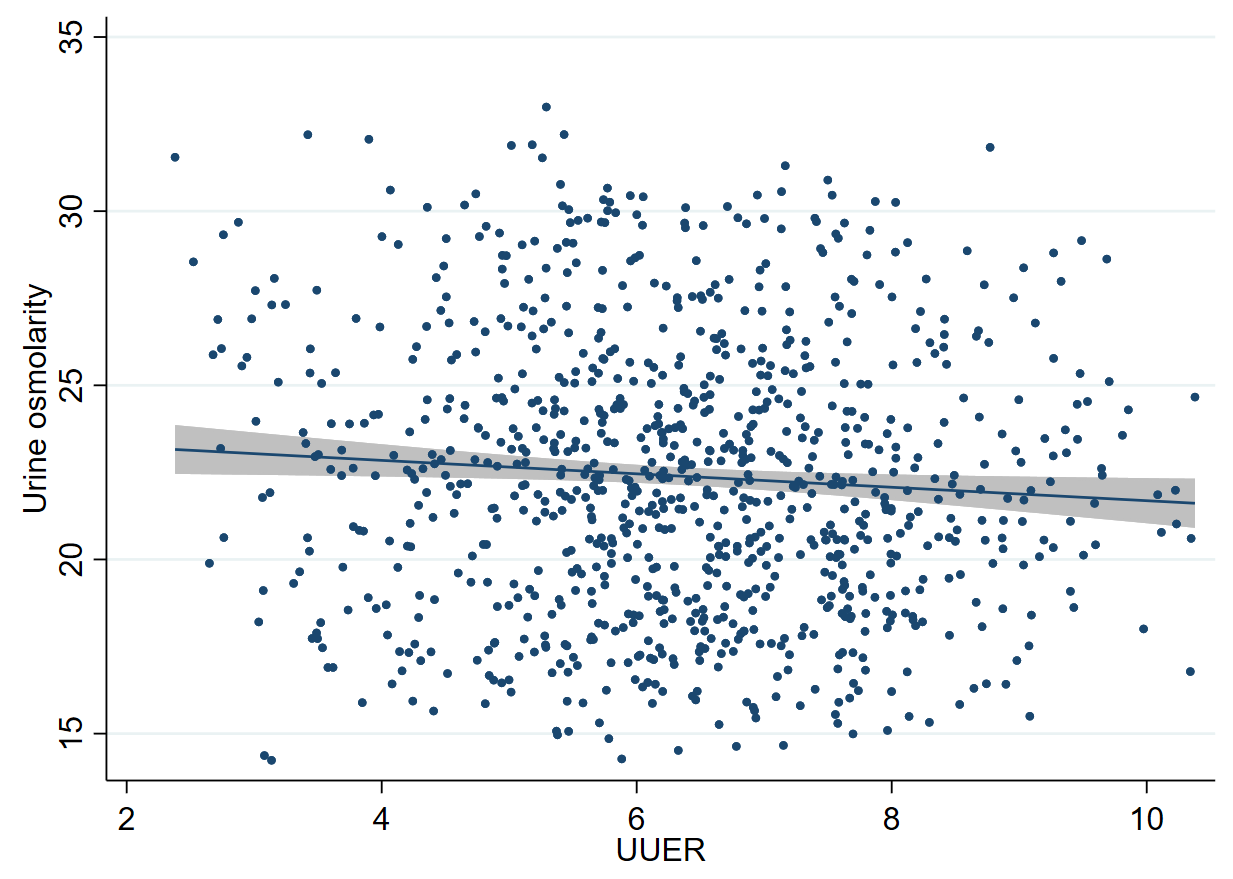


**STROBE checklist**.

STROBE Statement—Checklist of items that should be included in reports of ***cross-sectional studies***

|  | Item No | Recommendation | Page No |
| --- | --- | --- | --- |
| **Title and abstract** | 1 | (*a*) Indicate the study’s design with a commonly used term in the title or the abstract | 3 |
|  |  | (*b*) Provide in the abstract an informative and balanced summary of what was done and what was found | 3 |
| Introduction | | | |
| Background/rationale | 2 | Explain the scientific background and rationale for the investigation being reported | 5-6 |
| Objectives | 3 | State specific objectives, including any prespecified hypotheses | 6 |
| Methods | | | |
| Study design | 4 | Present key elements of study design early in the paper | 6 |
| Setting | 5 | Describe the setting, locations, and relevant dates, including periods of recruitment, exposure, follow-up, and data collection | 6 |
| Participants | 6 | (*a*) Give the eligibility criteria, and the sources and methods of selection of participants | 6 |
| Variables | 7 | Clearly define all outcomes, exposures, predictors, potential confounders, and effect modifiers. Give diagnostic criteria, if applicable | 6-8 |
| Data sources/ measurement | 8* | For each variable of interest, give sources of data and details of methods of assessment (measurement). Describe comparability of assessment methods if there is more than one group | *6-8* |
| Bias | 9 | Describe any efforts to address potential sources of bias | 6-8 |
| Study size | 10 | Explain how the study size was arrived at | 6 |
| Quantitative variables | 11 | Explain how quantitative variables were handled in the analyses. If applicable, describe which groupings were chosen and why | 7-8 |
| Statistical methods | 12 | (*a*) Describe all statistical methods, including those used to control for confounding | 7-8 |
|  |  | (*b*) Describe any methods used to examine subgroups and interactions | 7-8 |
|  |  | (*c*) Explain how missing data were addressed | 7-8 |
|  |  | (*d*) If applicable, describe analytical methods taking account of sampling strategy | 7-8 |
|  |  | (*e*) Describe any sensitivity analyses | 7-8 |
| Results | | | |
| Participants | 13* | (a) Report numbers of individuals at each stage of study—eg numbers potentially eligible, examined for eligibility, confirmed eligible, included in the study, completing follow-up, and analysed | 9 |
|  |  | (b) Give reasons for non-participation at each stage | 9 |
|  |  | (c) Consider use of a flow diagram | 9 |
| Descriptive data | 14* | (a) Give characteristics of study participants (eg demographic, clinical, social) and information on exposures and potential confounders | 9 |
|  |  | (b) Indicate number of participants with missing data for each variable of interest | 9 |
| Outcome data | 15* | Report numbers of outcome events or summary measures | 9 |
| Main results | 16 | (*a*) Give unadjusted estimates and, if applicable, confounder-adjusted estimates and their precision (eg, 95% confidence interval). Make clear which confounders were adjusted for and why they were included | 9-11 |
|  |  | (*b*) Report category boundaries when continuous variables were categorized | 22 (table 1) |
|  |  | (*c*) If relevant, consider translating estimates of relative risk into absolute risk for a meaningful time period | NA |
| Other analyses | 17 | Report other analyses done—eg analyses of subgroups and interactions, and sensitivity analyses | 12 |
| Discussion | | | |
| Key results | 18 | Summarise key results with reference to study objectives | 12-15 |
| Limitations | 19 | Discuss limitations of the study, taking into account sources of potential bias or imprecision. Discuss both direction and magnitude of any potential bias | 15 |
| Interpretation | 20 | Give a cautious overall interpretation of results considering objectives, limitations, multiplicity of analyses, results from similar studies, and other relevant evidence | 12-15 |
| Generalisability | 21 | Discuss the generalisability (external validity) of the study results | 15 |
| Other information | | | |
| Funding | 22 | Give the source of funding and the role of the funders for the present study and, if applicable, for the original study on which the present article is based | 2 |

*Give information separately for exposed and unexposed groups.

**Note:** An Explanation and Elaboration article discusses each checklist item and gives methodological background and published examples of transparent reporting. The STROBE checklist is best used in conjunction with this article (freely available on the Web sites of PLoS Medicine at http://www.plosmedicine.org/, Annals of Internal Medicine at http://www.annals.org/, and Epidemiology at http://www.epidem.com/). Information on the STROBE Initiative is available at www.strobe-statement.org.
